# Intelligence and Creativity in Problem Solving: The Importance of Test Features in Cognition Research

**DOI:** 10.3389/fpsyg.2017.00134

**Published:** 2017-02-06

**Authors:** Saskia Jaarsveld, Thomas Lachmann

**Affiliations:** Center for Cognitive Science, Cognitive and Developmental Psychology Unit, University of KaiserslauternKaiserslautern, Germany

**Keywords:** creative cognition, cognitive neuroscience, creative reasoning, problem space, knowledge domain

## Abstract

This paper discusses the importance of three features of psychometric tests for cognition research: construct definition, problem space, and knowledge domain. Definition of constructs, e.g., intelligence or creativity, forms the theoretical basis for test construction. Problem space, being well or ill-defined, is determined by the cognitive abilities considered to belong to the constructs, e.g., convergent thinking to intelligence, divergent thinking to creativity. Knowledge domain and the possibilities it offers cognition are reflected in test results. We argue that (a) comparing results of tests with different problem spaces is more informative when cognition operates in both tests on an identical knowledge domain, and (b) intertwining of abilities related to both constructs can only be expected in tests developed to instigate such a process. Test features should guarantee that abilities can contribute to self-generated *and* goal-directed processes bringing forth solutions that are both new and applicable. We propose and discuss a test example that was developed to address these issues.

The *definition* of the construct a test is to measure is most important in test construction and application, because cognitive processes reflect the possibilities a task offers. For instance, a test constructed to assess intelligence will operationalize the definition of this construct, being, in short, finding the correct answer. Also, the definition of a construct becomes important when selecting tests for the confirmation of a specific hypothesis. One can only find confirmation for a hypothesis if the chosen task instigates the necessary cognitive operations. For instance, in trying to confirm the assumed intertwining of certain cognitive abilities (e.g., convergent thinking and divergent thinking), tasks should be applied that have shown to yield the necessary cognitive process.

The second test feature, *problem space*, determines the degrees of freedom cognition has to its disposal in solving a problem. For instance, cognition will go through a wider search path when problem constraints are less well defined and, consequently, data will differ accordingly.

The third test feature, *knowledge domain*, is important when comparing results from two different tests. When tests differ in problem space, it is not advisable they should differ in knowledge domain. For instance, when studying the differences in cognitive abilities between tests constructed to asses convergent thinking (mostly defined problem space) and divergent thinking (mostly ill-defined problem space), in general test practice, both tests also differ in knowledge domain. Hence, data will reflect cognition operating not only in different problem spaces, but also operating on different knowledge domains, which makes the interpretation of results ambiguous.

The proposed approach for test development and test application holds the promise of, firstly, studying cognitive abilities in different problem spaces while operating on an identical knowledge domain. Although cognitions’ operations have been studied extensively and superbly in both contexts separately, they have rarely been studied in test situations where one or the other test feature is controlled for. The proposed approach also presents a unique method for studying thinking processes in which cognitive abilities intertwine. On the basis of defined abilities, tasks can be developed that have a higher probability of yielding the hypothesized results.

The construct of intelligence is defined as the ability to produce the single best (or correct) answer to a clearly defined question, such as a proof to a theorem (Simon, 1973). It may also be seen as a domain-general ability (*g*-factor; Spearman, 1904; Cattell, 1967) that has much in common with meta cognitive functions, such as metacognitive knowledge, metacognitive monitoring, and metacognitive control (Saraç et al., 2014).

The construct of creativity, in contrast, is defined as the ability to innovate and move beyond what is already known (Wertheimer, 1945/1968; Ghiselin, 1952/1985; Vernon, 1970). In other words, it emphasizes the aspect of innovation. This involves the ability to consider things from an uncommon perspective, transcend the old order (Ghiselin, 1952/1985; Chi, 1997; Ward, 2007), and explore loosely associated ideas (Guilford, 1950; Mednick, 1962; Koestler, 1964; Gentner, 1983; Boden, 1990; Christensen, 2007). Creativity could also be defined as the ability to generate a solution to problems with ill-defined problem spaces (Wertheimer, 1945/1968; Getzels and Csikszentmihalyi, 1976). In this sense it involves the ability to identify problematic aspects of a given situation (Ghiselin, 1952/1985) and, in a wider sense, the ability to define completely new problems (Getzels, 1975, 1987).

Guilford (1956) introduced the constructs of *convergent thinking* and *divergent thinking* abilities. Both thinking abilities are important because they allow us insights in human problem solving. On the basis of their definitions convergent and divergent thinking help us to structurally study human cognitive operations in different situations and over different developmental stages. Convergent thinking is defined as the ability to apply conventional and logical search, recognition, and decision-making strategies to stored information in order to produce an already known answer (Cropley, 2006). Divergent thinking, by contrast, is defined as the ability to produce new approaches and original ideas by forming unexpected combinations from available information and by applying such abilities as semantic flexibility, and fluency of association, ideation, and transformation (Guilford, 1959, as cited in Cropley, 2006, p. 1). Divergent thinking brings forth answers that may never have existed before and are often novel, unusual, or surprising (Cropley, 2006).

Guilford (1967) introduced convergent and divergent thinking as part of a set of five operations that apply in his Structure of Intellect model (SOI model) on six products and four kinds of content, to produce 120 different factors of cognitive abilities. With the SOI model Guilford wanted to give the construct of intelligence a comprehensive model. He wanted the model to include all aspects of intelligence, many of which had been seriously neglected in traditional intelligence testing because of a persistent adherence to the belief in Spearman’s *g* (Guilford, 1967, p. vii). Hence, Guilford envisaged cognition to embrace, among other abilities, both convergent and divergent thinking abilities. After these new constructs were introduced and defined, tests for convergent and divergent thinking emerged. Despite the fact that Guilford reported significant loadings of tests for divergent production on tests constructed to measure convergent production (Guilford, 1967, p. 155), over the years, both modes of thinking were considered as separate identities where convergent thinking tests associated with intelligence and divergent thinking tests with creativity (Cropley, 2006; Shye and Yuhas, 2004). Even intelligence tests that assess aspects of intelligence that supposedly reflect creative abilities do not actually measure creativity (Kaufman, 2015).

The idea that both convergent and divergent thinking are important for solving problems, and that intelligence helps in the creative process, is not really new. In literature we find models of the creative process that define certain stages to convergent and divergent thinking; the stages of purposeful preparation at the start and those of critical verification at the end of the process, respectively (Wallas, 1926; Webb Young, 1939/2003). In this view, divergent thinking enables the generation of new ideas whereas the exploratory activities of convergent thinking enable the conversion of ideas into something new and appropriate (Cropley and Cropley, 2008).

We argue that studying the abilities of divergent and convergent thinking in isolation does not suffice to give us complete insight of all possible aspects of human problem solving, its constituent abilities and the structure of its processes. Processes that in a sequence of thoughts and actions lead to novel and adaptive productions (Lubart, 2001) are more demanding of cognition for understanding the situation at hand and planning a path to a possible solution, than abilities involved in less complex situations (Jaušovec, 1999). Processes that yield self-generated *and* goal-directed thought are the most complex cognitive processes that can be studied (Beaty et al., 2016). Creative cognition literature is moving toward the view that especially in those processes that yield original *and* appropriate solutions within a specific context, convergent *and* divergent abilities intertwine (Cropley, 2006; Ward, 2007; Gabora, 2010).

The approach of intertwining cognitive abilities is also developed within cognitive neuroscience by focusing on the intertwining of brain networks (Beaty et al., 2016). In this approach divergent thinking relates to the *default* brain network. This network operates in defocused or *associative* mode of thought yielding spontaneous and self-generated cognition (Beaty et al., 2015). Convergent thinking relates to the *executive* control network operating in focused or *analytic* modes of thought, yielding updating, shifting, and inhibition (Benedek et al., 2014). Defocused attention theory (Mendelssohn, 1976) states that less creative individuals operate with a more focused attention than do creative individuals. This theory argues that e.g., attending to two things at the same time, might result in one analogy, while attending to four things might yield six analogies (Martindale, 1999).

In the process of shifting back and forth along the spectrum between associative and analytic modes of thinking, the fruits of associative thought become ingredients for analytic thought processes, and vice versa (Gabora, 2010). In this process, mental imagery is involved as one sensory aspect of the human ability to gather and process information (Jung and Haier, 2013). Mental imagery is fed by scenes in the environment that provide crucial visual clues for creative problem solving and actuates the need for sketching (Verstijnen et al., 2001).

Creative problem solving processes often involve an interactive relationship between imagining, sketching, and evaluating the result of the sketch (van Leeuwen et al., 1999). This interactive process evolves within a type of imagery called “visual reasoning” where forms and shapes are manipulated in order to specify the configurations and properties of the design entities (Goldschmidt, 2013). The originality of inventions is predicted by the application of visualization, whereas their practicality is predicted by the vividness of imagery (Palmiero et al., 2015). Imaginative thought processes emerge from our conceptual knowledge of the world that is represented in our semantic memory system. In constrained divergent thinking, the neural correlates of this semantic memory system partially overlap with those of the creative cognition system (Abraham and Bubic, 2015).

Studies of convergent and divergent thinking abilities have yielded innumerable valuable insights on the cognitive and neurological aspects involved, e.g., reaction times, strategies, brain areas involved, mental representations, and short and long time memory components. Studies on the relationship between both constructs suggest that it is unlikely that individuals employ similar cognitive strategies when solving more convergent than more divergent thinking tasks (Jaušovec, 2000). However, to arrive at a quality formulation the creative process cannot do without the application of both, convergent and divergent thinking abilities (e.g., Kaufmann, 2003; Runco, 2003; Sternberg, 2005; Dietrich, 2007; Cropley and Cropley, 2008; Silvia et al., 2013; Jung, 2014).

When it is our aim to study the networks addressed by the intertwining of convergent and divergent thinking processes that are considered to operate when new, original, and yet appropriate solutions are generated, then traditional thinking tests like intelligence tests and creativity tests are not appropriate; they yield processes related to the definition of one or the other type of construct.

## Creative Reasoning Task

According to the new insights gained in cognition research, we need tasks that are developed with the aim to instigate precisely the kind of thinking processes we are looking for. Tasks should also provide a method of scoring independently the contribution of convergent and divergent thinking. As one possible solution for such tasks we present the *Creative Reasoning Task* (CRT; Jaarsveld, 2007; Jaarsveld et al., 2010, 2012, 2013).

The CRT presents participants with an empty 3 × 3 matrix and asks them to fill it out, as original and complex as possible, by creating components and the relationships that connect them. The created matrix can, in principle, be solved by another person. The creation of components is entirely free, as is the generation of the relationships that connects them into a completed pattern. Created matrices are scored with two sub scores; *Relations*, which scores the logical complexity of a matrix and is, therefore, considered a measure for convergent thinking, and *Components and Specifications*, which scores the originality, fluency, and flexibility and, therefore, is considered an indication for divergent thinking (for a more detailed description of the score method, see Appendix 1 in Supplementary Material).

Psychometric studies with the CRT showed, firstly, that convergent and divergent thinking abilities apply within this task and can be assessed independently. The CRT sub score Relations correlated with the Standard Progressive Matrices test (SPM) and the CRT sub score Components and Specifications correlated with a standard creativity test (TCT–DP, Test of Creative Thinking–Drawing Production; Urban and Jellen, 1995; Jaarsveld et al., 2010, 2012, 2013). Studies further showed that, although a correlation was observed for the intelligence and creativity test scores, no correlation was observed between the CRT sub scores relating to intelligent and creative performances (Jaarsveld et al., 2012, 2013; for further details about the CRT’s objectivity, validity, and reliability, see Appendix 2 in Supplementary Material).

Reasoning in creative thinking can be defined as the involvement of executive/convergent abilities in the inhibition of ideas and the updating of information (Benedek et al., 2014). Jung (2014) describes a dichotomy for cognitive abilities with at one end the dedicated system that relies on explicit and conscious knowledge and at the other end the improvisational system that relies more upon implicit or unconscious knowledge systems. The link between explicit and implicit systems can actually be traced back to Kris’ psychoanalytic approach to creativity dating from the 1950s. The implicit system refers to Kris’ primary process of adaptive regression, where unmodulated thoughts intrude into consciousness; the explicit system refers to the secondary process, where the reworking and transformation of primary process material takes place through reality-oriented and ego-controlled thinking (Sternberg and Lubart, 1999). The interaction between explicit and implicit systems can be seen to form the basis of creative reasoning, i.e., the cognitive ability to solve problems in an effective and adaptive way. This interaction evolved as a cognitive mechanism when human survival depended on finding effective solutions to both common and novel problem situations (Gabora and Kaufman, 2010). Creative reasoning solves that minority of problems that are unforeseen and yet of high adaptability (Jung, 2014).

Hence, common tests are insufficient when it comes to solving problems that are unforeseen and yet of high adaptability, because they present problems that are either unforeseen and measure certain abilities contained in the construct of creativity or they address adaptability and measure certain abilities contained in the construct of intelligence. The CRT presents participants with a problem that they could not have foreseen; the form is blank and offers no stimuli. All tests, even creativity tests, present participants with some kind of stimuli. The CRT addresses adaptability; to invent from scratch a coherent structure that can be solved by another person, like creating a crossword puzzle. Problems, that are unforeseen *and* of high adaptability, are solved by the application of abilities from both constructs.

## Neuroscience of Creative Cognition

Studies in neuroscience showed that cognition operating in ill-defined problem space not only applies divergent thinking but also benefits from additional convergent operations (Gabora, 2010; Jung, 2014). Understanding creative cognition may be advanced when we study the flow of information among brain areas (Jung et al., 2010).

In a cognitive neuroscience study with the CRT we focused on the cognitive process evolving within this task. Participants performed the CRT while EEG alpha activity was registered. EEG alpha synchronization in frontal areas is understood as an indication of top-down control (Cooper et al., 2003). When observed in frontal areas, for divergent and convergent thinking tasks, it may not reflect a brain state that is specific for creative cognition but could be attributed to the high processing demands typically involved in creative thinking (Benedek et al., 2011). Top-down control, relates to volitionally focusing attention to task demands (Buschman and Miller, 2007). That this control plays a role in tasks with an ill-defined problem space showed when electroencephalography (EEG) alpha synchronization was stronger for individuals engaged in creative ideation tasks compared to an intelligence related tasks (Fink et al., 2007, 2009; Fink and Benedek, 2014). This activation was also found for the CRT; task related alpha synchronization showed that convergent thinking was integrated in the divergent thinking processes. Analyzes of the stages in the CRT process showed that this alpha synchronization was especially visible at the start of the creative process at prefrontal and frontal sites when information processing was most demanding, i.e., due to multiplicity of ideas, and it was visible at the end of the process, due to narrowing down of alternatives (Jaarsveld et al., 2015).

A functional magnetic resonance imaging (fMRI) study (Beaty et al., 2015) with a creativity task in which cognition had to meet specific constraints, showed the networks involved. The default mode network which drives toward abstraction and metaphorical thinking and the executive control network driving toward certainty (Jung, 2014). Control involves not only maintenance of patterns of activity that represent goals and the means to achieve those (Miller and Cohen, 2001), but also their voluntary suppression when no longer needed, as well as the flexible shift between different goals and mental sets (Abraham and Windmann, 2007). Attention can be focused volitionally by top-down signals derived from task demands and automatically by bottom-up signals from salient stimuli (Buschman and Miller, 2007). Intertwining between top-down and bottom-up attention processes in creative cognition ensures a broadening of attention in free associative thinking (Abraham and Windmann, 2007).

These studies support and enhance the findings of creative cognition research in showing that the generation of original and applicable ideas involves an intertwining between different abilities, networks, and attention processes.

## Problem Space

A problem space is an abstract representation, in the mind of the problem solver, of the encountered problem and of the asked for solution (Simon and Newell, 1971; Simon, 1973; Hayes and Flowers, 1986; Kulkarni and Simon, 1988; Runco, 2007). The space that comes with a certain problem can, according to the constraints that are formulated for the solution, be labeled well-defined or ill-defined (Simon and Newell, 1971). Consequently, the original problems are labeled closed and open problems, respectively (Jaušovec, 2000).

A problem space contains all possible states that are accessible to the problem solver from the *initial state*, through iterative application of *transformation rules*, to the *goal state* (Newell and Simon, 1972; Anderson, 1983). The initial state presents the problem solver with a task description that defines which requirements a solution has to answer. The goal state represents the solution. The proposed solution is a product of the application of transformation rules (algorithms and heuristics) on a series of successive intermediate solutions. The proposed solution is also a product of the iterative evaluations of preceding solutions and decisions based upon these evaluations (Boden, 1990; Gabora, 2002; Jaarsveld and van Leeuwen, 2005; Goldschmidt, 2014). Whether all possible states need to be passed through depends on the problem space being well or ill-defined and this, in turn, depends on the character of the task descriptions.

When task descriptions clearly state which requirements a solution has to answer then the inferences made will show little idiosyncratic aspects and will adhere to the task constraints. As a result, fewer options for alternative paths are open to the problem solver and search for a solution evolves in a well-defined space. Vice versa, when task or problem descriptions are fuzzy and under specified, the problem solver’s inferences are more idiosyncratic; the resulting process will evolve within an ill-defined space and will contain more generative-evaluative cycles in which new goals are set, and the cycle is repeated (Dennett, 1978, as cited in Gabora, 2002, p. 126).

Tasks that evolve in defined problem space are, e.g., traditional intelligence tests (e.g., Wechsler Adult Intelligence Scale, WAIS; and SPM, Raven, 1938/1998). The above tests consist of different types of questions, each testing a different component of intelligence. They are used in test practice to assess reasoning abilities in diverse domains, such as, abstract, logical, spatial, verbal, numerical, and mathematical domains. These tests have clearly stated task descriptions and each item has one and only one correct solution that has to be generated from memory or chosen from a set of alternatives, like in multiple choice formats. Tests can be constructed to assess crystallized or fluid intelligence. Crystallized intelligence represents abilities acquired through learning, practice, and exposure to education, while fluid intelligence represents a more basic capacity that is valuable to reasoning and problem solving in contexts not necessarily related to school education (Carroll, 1982).

Tasks that evolve in ill-defined problem space are, e.g., standard creativity tests. These types of test ask for a multitude of ideas to be generated in association with a given item or situation (e.g., “think of as many titles for this story”). Therefore, they are also labeled as divergent thinking test. Although they assess originality, fluency, flexibility of responses, and elaboration, they are not constructed, however, to score appropriateness or applicability. Divergent thinking tests assess one limited aspect of what makes an individual creative. Creativity depends also on variables like affect and intuition; therefore, divergent thinking can only be considered an indication of an individual’s creative *potential* (Runco, 2008). More precisely, divergent thinking explains just under half of the variance in adult creative potential, which is more than three times that of the contribution of intelligence (Plucker, 1999, p. 103). Creative *achievement*, by contrast, is commonly assessed by means of self-reports such as biographical questionnaires in which participants indicate their achievement across various domains (e.g., literature, music, or theater).

Studies with the CRT showed that problem space differently affects processing of and comprehension of relationships between components. Problem space did not affect the ability to *process* complex information. This ability showed equal performance in well and ill-defined problem spaces (Jaarsveld et al., 2012, 2013). However, problem space did affect the *comprehension* of relationships, which showed in the different frequencies of relationships solved and created (Jaarsveld et al., 2010, 2012). Problem space also affected the neurological activity as displayed when individuals solve open or closed problems (Jaušovec, 2000).

Problem space further affected trends over grade levels of primary school children for relationships solved in well-defined and applied in ill-defined problem space. Only one of the 12 relationships defined in the CRT, namely Combination, showed an increase with grade for both types of problem spaces (Jaarsveld et al., 2013). In the same study, cognitive development in the CRT showed in the shifts of preference for a certain relationship. These shifts seem to correspond to Piaget’s developmental stages (Piaget et al., 1977; Siegler, 1998) which are in evidence in the CRT, but not in the SPM (Jaarsveld et al., 2013).

## Design Problems

A sub category of problems with an ill-defined problem space are represented by design problems. In contrast to divergent thinking tasks that ask for the generation of a multitude of ideas, in design tasks interim ideas are nurtured and incrementally developed until they are appropriate for the task. Ideas are rarely discarded and replaced with new ideas (Goel and Pirolli, 1992). The CRT could be considered a design problem because it yields (a) one possible solution and (b) an iterative thinking process that involves the realization of a vague initial idea. In the CRT a created matrix, which is a closed problem, is created within an ill-defined problem space. Design problems can be found, e.g., in engineering, industrial design, advertising, software design, and architecture (Sakar and Chakrabarti, 2013), however, they can also be found in the arts, e.g., poetry, sculpting, and dance geography.

These complex problems are partly determined by unalterable needs, requirements and intentions but the major part of the design problem is undetermined (Dorst, 2004). This author points out that besides containing an original and a functional value, these types of problems contain an aesthetic value. He further states that the interpretation of the design problem and the creation and selection of possible suitable solutions can only be decided during the design process on the basis of proposals made by the designer.

In design problems the generation stage may be considered a divergent thinking process. However, not in the sense that it moves in multiple directions or generates multiple possibilities as in a divergent thinking tests, but in the sense that it unrolls by considering an initially vague idea from different perspectives until it comes into focus and requires further processing to become viable. These processes can be characterized by a set of invariant features (Goel and Pirolli, 1992), e.g., *structuring. iteration*, and *coherence*.

Structuring of the initial situation is required in design processes before solving can commence. The problem contains little structured and clear information about its initial state and about the requirements of its solution. Therefore, design problems allow or even require re-interpretation of transformation rules; for instance, rearranging the location of furniture in a room according to a set of desirable outcomes. Here one uncovers implicit requirements that introduce a set of new transformations and/or eliminate existing ones (Barsalou, 1992; Goel and Pirolli, 1992) or, when conflicting requirements arise, one creates alternatives and/or introduces new trade-offs between the conflicting constraints (Yamamoto et al., 2000; Dorst, 2011).

A second aspect of design processes is their iterative character. After structuring and planning a vague idea emerges, which is the result of the merging of memory items. A vague idea is a cognitive structure that, halfway the creative process is still ill defined and, therefore, can be said to exist in a state of potentiality (Gabora and Saab, 2011). Design processes unroll in an iterative way by the inspection and adjustment of the generated ideas (Goldschmidt, 2014). New meanings are created and realized while the creative mind imposes its own order and meaning on the sensory data and through creative production furthers its own understanding of the world (Arnheim, 1962/1974, as cited in Grube and Davis, 1988, pp. 263–264).

A third aspect of design processes is coherence. Coherence theories characterize coherence in, for instance, philosophical problems and psychological processes, in terms of maximal satisfaction of multiple constraints and compute coherence by using, a.o., connectionist algorithms (Thagard and Verbeurgt, 1998). Another measure of coherence is characterized as continuity in design processes. This measure was developed for a design task (Jaarsveld and van Leeuwen, 2005) and calculated by the occurrence of a given pair of objects in a sketch, expressed as a percentage of all the sketches of a series. In a series of sketches participants designed a logo for a new soft drink. Design series strong in coherence also received a high score for their final design, as assessed by professionals in various domains. Indicating that participants with a high score for the creative quality of their final sketch seemed better in assessing their design activity in relation to the continuity in the process and, thereby, seemed better in navigating the ill-defined space of a design problem (Jaarsveld and van Leeuwen, 2005). In design problems the quality of cognitive production depends, in part, on the abilities to reflect on one’s own creative behavior (Boden, 1996) and to monitor how far along in the process one is in solving it (Gabora, 2002). Hence, design problems are especially suited to study more complex problem solving processes.

## Knowledge Domain

Knowledge domain represents disciplines or fields of study organized by general principles, e.g., domains of various arts and sciences. It contains accumulated knowledge that can be divided in diverse content domains, and the relevant algorithms and heuristics. We also speak of knowledge domains when referring to, e.g., visuo-spatial and verbal domains. This latter differentiation may refer to the method by which performance in a certain knowledge domain is assessed, e.g., a visuo-spatial physics task that assesses the content domain of the workings of mass and weights of objects.

In comparing tests results, we should keep in mind that apart from reflecting cognitive processes evolving in different problem spaces, the results also arise from cognition operating on different knowledge domains. We argue that, the still contradictory and inconclusive discussion about the relationship between intelligence and creativity (Silvia, 2008), should involve the issue of knowledge domain.

Intelligence tests contain items that pertain to, e.g., verbal, abstract, mechanical and spatial reasoning abilities, while their content mostly operates on knowledge domains that are related to contents contained in school curricula. Items of creativity tests, by contrast, pertain to more idiosyncratic knowledge domains, their contents relating to associations between stored personal experiences (Karmiloff-Smith, 1992). The influence of knowledge domain on the relationships between different test scores was already mentioned by Guilford (1956, p. 169). This author expected a higher correlation between scores from a typical intelligence test and a divergent thinking test than between scores from two divergent thinking tests because the former pair operated on identical information and the latter pair on different information.

Studies with the CRT showed that when knowledge domain is controlled for, the development of intelligence operating in ill-defined problem space does not compare to that of traditional intelligence but develops more similarly to the development of creativity (Welter et al., in press).

## Relationship Intelligence and Creativity

The Threshold theory (Guilford, 1967) predicts a relationship between intelligence and creativity up to approximately an intelligence quotient (IQ) level of 120 but not beyond (Lubart, 2003; Runco, 2007). Threshold theory was corroborated when creative potential was found to be related to intelligence up to certain IQ levels; however, the theory was refuted, when focusing on achievement in creative domains; it showed that creative achievement benefited from higher intelligence even at fairly high levels of intellectual ability (Jauk et al., 2013).

Distinguishing between subtypes of general intelligence known as fluent and crystallized intelligence (Cattell, 1967), Sligh et al. (2005) observed an inverse threshold effect with fluid IQ: a correlation with creativity test scores in the high IQ group but not in the average IQ group. Also creative achievement showed to be affected by fluid intelligence (Beaty et al., 2014). Intelligence, defined as fluid IQ, verbal fluency, and strategic abilities, showed a higher correlation with creativity scores (Silvia, 2008) than when defined as crystallized intelligence. Creativity tests, which involved convergent thinking (e.g., Remote Association Test; Mednick, 1962) showed higher correlations with intelligence than ones that involved only divergent thinking (e.g., the Alternate Uses Test; Guilford et al., 1978).

That the Remote Association test also involves convergent thinking follows from the instructions; one is asked, when presented with a stimulus word (e.g., table) to produce the first word one thinks of (e.g., chair). The word pair table–chair is a common association, more remote is the pair table–plate, and quite remote is table–shark. According to Mednick’s theory (a) all cognitive work is done essentially by combining or associating ideas and (b) individuals with more commonplace associations have an advantage in well-defined problem spaces, because the class of relevant associations is already implicit in the statement of the problem (Eysenck, 2003).

To circumvent the problem of tests differing in knowledge domain, one can develop out of one task a more divergent and a more convergent thinking task by asking, on the one hand, for the generation of original responses, and by asking, on the other hand, for more common responses (Jauk et al., 2012). By changing the instruction of a task, from convergent to divergent, one changes the constraints the solution has to answer and, thereby, one changes for cognition its freedom of operation (Razumnikova et al., 2009; Limb, 2010; Jauk et al., 2012). However, asking for more common responses is still a divergent thinking task because it instigates a generative and ideational process.

Indeed, studying the relationship between intelligence and creativity with knowledge domain controlled for yielded different results as defined in the Threshold theory. A study in which knowledge domain was controlled for showed, firstly, that intelligence is no predictor for the development of creativity (Welter et al., 2016). Secondly, that the relationship between scores of intelligence and creativity tests as defined under the Threshold theory was only observed in a small subset of primary school children, namely, female children in Grade 4 (Welter et al., 2016). We state that relating results of operations yielded by cognitive abilities performing in defined and in ill-defined problem spaces can only be informative when it is ensured that cognitive processes also operate on an identical knowledge domain.

## Intertwining of Cognitive Abilities

Eysenck (2003) observed that there is little justification for considering the constructs of divergent and convergent thinking in categorical terms in which one construct excludes the other. In processes that yield original and appropriate solutions convergent and divergent thinking both operate on the same large knowledge base and the underlying cognitive processes are not entirely dissimilar (Eysenck, 2003, p. 110–111).

Divergent thinking is especially effective when it is coupled with convergent thinking (Runco, 2003; Gabora and Ranjan, 2013). A design problem study (Jaarsveld and van Leeuwen, 2005) showed that divergent production was active throughout the design, as new meanings are continuously added to the evolving structure (Akin, 1986), and that convergent production was increasingly important toward the end of the process, as earlier productions are wrapped up and integrated in the final design. These findings are in line with the assumptions of Wertheimer (1945/1968) who stated that thinking within ill-defined problem space is characterized by two points of focus; one is to work on the parts, the other to make the central idea clearer.

Parallel to the discussion about the intertwining of convergent and divergent thinking abilities in processes that evolve in ill-defined problem space we find the discussion about how intelligence may facilitate creative thought. This showed when top-down cognitive control advanced divergent processing in the generation of original ideas and a certain measure of cognitive inhibition advanced the fluency of idea generation (Nusbaum and Silvia, 2011). Fluid intelligence and broad retrieval considered as intelligence factors in a structural equation study contributed both to the production of creative ideas in a metaphor generation task (Beaty and Silvia, 2013). The notion that creative thought involves top-down, executive processes showed in a latent variable analysis where inhibition primarily promoted the fluency of ideas, and intelligence promoted their originality (Benedek et al., 2012).

## Definitions of the Constructs Intelligence and Creativity

The various definitions of the constructs of intelligence and creativity show a problematic overlap. This overlap stems from the enormous endeavor to unanimously agree on valid descriptions for each construct. Spearman (1927), after having attended many symposia that aimed at defining intelligence, stated that “in truth, ‘intelligence’ has become a mere vocal sound, a word with so many meanings that finally it has none” (p. 14).

Intelligence is expressed in terms of adaptive, goal-directed behavior; and the subset of such behavior that is labeled “intelligent” seems to be determined in large part by cultural or societal norms (Sternberg and Salter, 1982). The development of the IQ measure is discussed by Carroll (1982): “Binet (around 1905) realized that intelligent behavior or mental ability can be ranged along a scale. Not much later, Stern (around 1912) noticed that, as chronological age increased, variation in mental age changes proportionally. He developed the IQ ratio, whose standard deviation would be approximately constant over chronological age if mental age was divided by chronological age. With the development of *multiple-factor-analyses* (Thurstone, around 1935) it could be shown that intelligence is not a simple unitary trait because at least seven somewhat independent factors of mental ability were identified.”

Creativity is defined as a combined manifestation of novelty and usefulness (Jung et al., 2010). Although it is identified with divergent thinking, and performance on divergent thinking tasks predicts, e.g., quantity of creative achievements (Torrance, 1988, as cited in Beaty et al., 2014) and quality of creative performance (Beaty et al., 2013), it cannot be identified uniquely with divergent thinking.

Divergent thinking often leads to highly original ideas that are honed to appropriate ideas by evaluative processes of critical thinking, and valuative and appreciative considerations (Runco, 2008). Divergent thinking tests should be more considered as estimates of creative problem solving potential rather than of actual creativity (Runco, 1991). Divergent thinking is not specific enough to help us understand what, exactly, are the mental processes—or the cognitive abilities—that yield creative thoughts (Dietrich, 2007).

Although current definitions of intelligence and creativity try to determine for each separate construct a unique set of cognitive abilities, analyses show that definitions vary in the degree to which each includes abilities that are generally considered to belong to the other construct (Runco, 2003; Jaarsveld et al., 2012). Abilities considered belonging to the construct of intelligence such as hypothesis testing, inhibition of alternative responses, and creating mental images of new actions or plans are also considered to be involved in creative thinking (Fuster, 1997, as cited in Colom et al., 2009, p. 215). The ability, for instance, to *evaluate*, which is considered to belong to the construct of intelligence and assesses the match between a proposed solution and task constraints, has long been considered to play a role in creative processes that goes beyond the mere generation of a series of ideas as in creativity tasks (Wallas, 1926, as cited in Gabora, 2002, p. 1; Boden, 1990).

The Geneplore model (Finke et al., 1992) explicitly models this idea; after stages in which objects are merely generated, follow phases in which an object’s utility is explored and estimated. The generation phase brings forth pre inventive objects, imaginary objects that are generated without any constraints in mind. In exploration, these objects are evaluated for their possible functionalities. In anticipating the functional characteristics of generated ideas, convergent thinking is needed to apprehend the situation, make evaluations (Kozbelt, 2008), and consider the consequences of a chosen solution (Goel and Pirolli, 1992). Convergent reasoning in creativity tasks invokes criteria of functionality and appropriateness (Halpern, 2003; Kaufmann, 2003), goal directedness and adaptive behavior (Sternberg, 1982), as well as the abilities of planning and attention. Convergent thinking stages may even require divergent thinking sub processes to identify restrictions on proposed new ideas and suggest requisite revision strategies (Mumford et al., 2007). Hence, evaluation, which is considered to belong to the construct of intelligence, is also functional in creative processes.

In contrast, the ability of *flexibility*, which is considered to belong to the construct of creativity and denotes an openness of mind that ensures the generation of ideas from different domains, showed, as a factor component for latent divergent thinking, a relationship with intelligence (Silvia, 2008). Flexibility was also found to play an important role in intelligent behavior where it enables us to do novel things smartly in new situations (Colunga and Smith, 2008). These authors studied children’s generalizations of novel nouns and concluded that if we are to understand human intelligence, we must understand the processes that make inventiveness. They propose to include the construct of flexibility within that of intelligence. Therefore, definitions of the constructs we are to measure affect test construction and the resulting data. However, an overlap between definitions, as discussed, yields a test diversity that makes it impossible to interpret the different findings across studies with any confidence (Arden et al., 2010). Also Kim (2005) concluded that because of differences in tests and administration methods, the observed correlation between intelligence and creativity was negligible. As the various definitions of the constructs of intelligence and creativity show problematic overlap, we propose to circumvent the discussion about which cognitive abilities are assessed by which construct, and to consider both constructs as being involved in one design process. This approach allows us to study the contribution to this process of the various defined abilities, without one construct excluding the other.

## Reasoning Abilities

The CRT is a psychometrical tool constructed on the basis of an alternative construct of human cognitive functioning that considers creative reasoning as a thinking process understood as the cooperation between cognitive abilities related to intelligent and creative thinking.

In generating relationships for a matrix, reasoning and more specifically the ability of rule invention is applied. The ability of rule invention could be considered as an extension of the sequence of abilities of rule learning, rule inference, and rule application, implying that creativity is an extension of intelligence (Shye and Goldzweig, 1999). According to this model, we could expect different results between a task assessing abilities of rule learning and rule inference, and a task assessing abilities of rule application. In two studies rule learning and rule inference was assessed with the RPM and rule application was assessed with the CRT. Results showed that from Grades 1 to 4, the frequencies of relationships applied did not correlate with those solved (Jaarsveld et al., 2010, 2012). Results showed that performance in the CRT allows an insight of cognitive abilities operating on relationships among components that differs from the insight based on performance within the same knowledge domain in a matrix solving task. Hence, reasoning abilities lead to different performances when applied in solving closed as to open problems.

We assume that reasoning abilities are more clearly reflected when one formulates a matrix from scratch; in the process of thinking and drawing one has, so to speak, to solve one’s own matrix. In doing so one explains to oneself the relationship(s) realized so far and what one would like to attain. Drawing is thinking aloud a problem and aids the designer’s thinking processes in providing some “talk-back” (Cross and Clayburn Cross, 1996). Explanatory activity enhances learning through increased depth of processing (Siegler, 2005). Analyzing explanations of examples given with physics problems showed that they clarify and specify the conditions and consequences of actions, and that they explicate tacit knowledge; thereby enhancing and completing an individual’s understanding of principles relevant to the task (Chi and VanLehn, 1991). Constraint of the CRT is that the matrix, in principle, can be solved by another person. Therefore, in a kind of inner explanatory discussion, the designer makes observations of progress, and uses evaluations and decisions to answer this constraint. Because of this, open problems where certain constraints have to be met, constitute a powerful mechanism for promoting understanding and conceptual advancement (Chi and VanLehn, 1991; Mestre, 2002; Siegler, 2005).

## Conclusion

Convergent and divergent thinking processes have been studied with a variety of intelligence and creativity tests, respectively. Relationships between performances on these tests have been demonstrated and a large number of research questions have been addressed. However, the fact that intelligence and creativity tests vary in the definition of their construct, in their problem space, and in their knowledge domain, poses methodological problems regarding the validity of comparisons of test results. When we want to focus on one cognitive process, e.g., intelligent thinking, and on its different performances in well or ill-defined problem situations, we need pairs of tasks that are constructed along identical definitions of the construct to be assessed, that differ, however, in the description of their constraints but are identical regarding their knowledge domain.

One such possible pair, the Progressive Matrices Test and the CRT was suggested here. The CRT was developed on the basis of *creative reasoning*, a construct that assumes the intertwining of intelligent and creativity related abilities when looking for original and applicable solutions. Matched with the Matrices test, results indicated that, besides similarities, intelligent thinking also yielded considerable differences for both problem spaces. Hence, with knowledge domain controlled, and only differences in problem space remaining, comparison of data yielded new results on intelligence’s operations. Data gathered from intelligence and creativity tests, whether they are performance scores or physiological measurements on the basis of, e.g., EEG, and fMRI methods, are reflections of cognitive processes performing on a certain test that was constructed on the basis of a certain definition of the construct it was meant to measure. Data are also reflections of the processes evolving within a certain problem space and of cognitive abilities operating on a certain knowledge domain.

Data can unhide brain networks that are involved in the performance of certain tasks, e.g., traditional intelligence and creativity tests, but data will always be related to the characteristics of the task. The characteristics of the task, such as problem space and knowledge domain originated at the construction of the task, and the construction, on its turn, is affected by the definition of the construct the task is meant to measure.

Here we present the CRT as one possible solution for the described problems in cognition research. However, for research on relationships among test scores other pairs of tests are imaginable, e.g., pairs of tasks operating on the same domain where one task has a defined problem space and the other one an ill-defined space. It is conceivable that pairs of test could operate, besides on the domain of mathematics, on content of e.g., visuo-spatial, verbal, and musical domains. Pairs of test have been constructed by changing the instruction of a task; instructions instigated a more convergent or a more a divergent mode of response (Razumnikova et al., 2009; Limb, 2010; Jauk et al., 2012; Beaty et al., 2013).

The CRT involves the creation of components and their relationships for a 3 × 3 matrix. Hence, matrices created in the CRT are original in the sense that they all bear individual markers and they are applicable in the sense, that they can, in principle, be solved by another person. We showed that the CRT instigates a real design process; creators’ cognitive abilities are wrapped up in a process that should produce a closed problem within an ill-defined problem space.

For research on the relationship among convergent and divergent thinking, we need pairs of test that differ in the *problem spaces* related to each test but are identical in the *knowledge domain* on which cognition operates. The test pair of RPM and CRT provides such a pair. For research on the intertwining of convergent and divergent thinking, we need tasks that measure more than tests assessing each construct alone. We need tasks that are developed on the *definition* of intertwining cognitive abilities; the CRT is one such test.

Hence, we hope to have sufficiently discussed and demonstrated the importance of the three test features, construct definition, problem space, and knowledge domain, for research questions in creative cognition research.

## Author Contributions

All authors listed, have made substantial, direct and intellectual contribution to the work, and approved it for publication.

## Conflict of Interest Statement

The authors declare that the research was conducted in the absence of any commercial or financial relationships that could be construed as a potential conflict of interest.
