# Supplementary material for: Intelligence and Creativity in Problem Solving: The Importance of Test Features in Cognition Research
Source: Front Psychol. 2017 Feb 6;8:134. doi: 10.3389/fpsyg.2017.00134 (PMC5292426; doi:10.3389/fpsyg.2017.00134)
Supplement: Supplementary file 1 [file Data_Sheet_1.docx]

**Appendix 1**. CRT Score Method. The CRT sub score *Relations* applies twelve relationships, e.g.: Continuous Pattern, Groups of Three Components, and Combination (for a detailed description of relationships see Jaarsveld et al., 2010, 2012). *Relations* is a summation over the values that are assigned to the components belonging to a relationship. Therefore, it can assign scores not only to complete but also to incomplete matrices. Weights are added to the sub score of a relationship to express its complexity. This sub score can be considered an indication for convergent thinking. The sub score *Components and Specification*s scores components’ type (figurative, geometrical), category (square, triangle, circle, etc.), and applied transformations (size, orientation, number, and location), only as long as the components’ transformations do not express a relationship. This sub score can be considered an indication for divergent thinking.

**Appendix 2**. Test Objectivity, Validity, and Reliability. Objectivity: Although the CRT (Creative Reasoning Task*,* Jaarsveld, 2007; Jaarsveld, Lachmann, Hamel, & van Leeuwen, 2010; Jaarsveld, Lachmann, & van Leeuwen, 2012; Jaarsveld, Lachmann, & van Leeuwen, 2013) is not a published test yet, we obtained inter rater correlations on two different sets of data with Cohen’s Kappa, К, ranging from К=0.95 to К=0.93 (*N*_1_=95, *N*_2_=29) (Jaarsveld, Lachmann, Hamel, and van Leeuwen, 2010). Validity: CRT scores for convergent thinking correlated with a standard intelligence test (SPM, Standard Progressive Matrices test; Raven, 1938/1998), *r*=.213, *p*<.01) and CRT divergent thinking scores correlated with a standard creativity test (TCT–DP, Test of Creative Thinking-Drawing Production; Urban & Jellen, 1995), *r*=.153, *p*<.05, both *N*=205 (Jaarsveld et al., 2012). Reliability: We also obtained identical developmental scores in two groups, Spearman Rank correlations of frequencies applied over grade, p < .05 (N_1_ = 511, 4-12y old; N_2_= 205, 6-10y old) (Jaarsveld et al., 2013).
